# NK1.1^−^ CD4^+^ NKG2D^+^ T cells suppress DSS‐induced colitis in mice through production of TGF‐β

**DOI:** 10.1111/jcmm.13072

**Published:** 2017-02-22

**Authors:** Xingxing Qian, Chunxia Hu, Sen Han, Zhijie Lin, Weiming Xiao, Yanbing Ding, Yu Zhang, Li Qian, Xiaoqing Jia, Guoqiang Zhu, Weijuan Gong

**Affiliations:** ^1^ Department of ImmunologySchool of MedicineYangzhou UniversityYangzhouChina; ^2^ Jiangsu Key Laboratory of Integrated Traditional Chinese and Western Medicine for Prevention and Treatment of Senile DiseasesYangzhouChina; ^3^ Jiangsu Key Laboratory of ZoonosisYangzhouChina; ^4^ Department of GastroenterologyThe Second Clinical Medical CollegeYangzhou UniversityYangzhouChina; ^5^ Jiangsu Co‐innovation Center for Prevention and Control of Important Animal Infectious Diseases and ZoonosesYangzhouChina

**Keywords:** CD4, NKG2D, colitis, NK1.1, regulatory

## Abstract

CD4^+^ NKG2D^+^ T cells are associated with tumour, infection and autoimmune diseases. Some CD4^+^ NKG2D^+^ T cells secrete IFN‐γ and TNF‐α to promote inflammation, but others produce TGF‐β and FasL to facilitate tumour evasion. Here, murine CD4^+^ NKG2D^+^ T cells were further classified into NK1.1^−^ CD4^+^ NKG2D^+^ and NK1.1^+^ CD4^+^ NKG2D^+^ subpopulations. The frequency of NK1.1^−^ CD4^+^ NKG2D^+^ cells decreased in inflamed colons, whereas more NK1.1^+^ CD4^+^ NKG2D^+^ cells infiltrated into colons of mice with DSS‐induced colitis. NK1.1^−^ CD4^+^ NKG2D^+^ cells expressed TGF‐β and FasL without secreting IFN‐γ, IL‐21 and IL‐17 and displayed no cytotoxicity. The adoptive transfer of NK1.1^−^ CD4^+^ NKG2D^+^ cells suppressed DSS‐induced colitis largely dependent on TGF‐β. NK1.1^−^ CD4^+^ NKG2D^+^ cells did not expressed Foxp3, CD223 (LAG‐3) and GITR. The subpopulation was distinct from NK1.1^+^ CD4^+^ NKG2D^+^ cells in terms of surface markers and RNA transcription. NK1.1^−^ CD4^+^ NKG2D^+^ cells also differed from Th2 or Th17 cells because the former did not express GATA‐3 and ROR‐γt. Thus, NK1.1^−^ CD4^+^ NKG2D^+^ cells exhibited immune regulatory functions, and this T cell subset could be developed to suppress inflammation in clinics.

## Introduction

NKG2D is an activating receptor expressed on natural killer (NK) cells, CD8^+^ T, γδ T and NKT cells as well as on activated macrophages. Ligands of human NKG2D include MHC class I chain‐related protein A/B (MICA/MICB) and UL16‐binding proteins (ULBPs). Mouse NKG2D ligands include retinoic acid early induced transcript‐1 (RAE‐1α, RAE‐1β, RAE‐1γ, RAE‐1δ, RAE‐1ε), H60 and murine ULBP‐like transcript 1 (MULT‐1) 1, 2. NKG2D ligation can stimulate NK cells and costimulate CD8^+^ T cells. NKG2D in association with its adaptor molecules, namely DAP10 or DAP12, transduces signalling by the activation of phosphatidylinositol 3‐kinase (PI3K), resulting in cell activation, survival, cytoskeletal rearrangement and release of cytokines and cytotoxic granules 3, 4.

CD4^+^ NKG2D^+^ T cells are associated with inflammatory diseases, such as rheumatoid arthritis (RA) 5, 6, granulomatosis with polyangiitis 7, Crohn's disease 8, 9, 10, multiple sclerosis 11 and infection by human cytomegalovirus (CMV) 12. Patients with cervical intraepithelial neoplasia grade 1 13, 14, colon cancer 15 and melanoma who were pre‐treated with a multityrosine kinase inhibitor (sorafenib) also exhibit enhanced frequencies of CD4^+^ NKG2D^+^ T cells 16. These CD4^+^ NK2D^+^ T cells exhibit Th1‐like properties in tissues because of the produced IFN‐γ, TNF‐α and cytolytic granules. IL‐15 of the microenvironment either *in cis* or *in trans* form contributes to the induction of CD4^+^ NKG2D^+^ T cell subset 5, 7, 16.

CD4^+^ NKG2D^+^ T cell population, which is associated in regulatory activities, is normally found in healthy individuals; CD4^+^ NKG2D^+^ T cell population is inversely correlated with disease severity in patients with juvenile‐onset systemic lupus, suggesting that CD4^+^ NKG2D^+^ T cells acts in regulation rather than inflammation 17. Furthermore, studies of patients with different malignancies indicated that a large proportion of CD4^+^ NKG2D^+^ T cells with regulatory activity is largely dependent on FasL and TGF‐β; hence, this T cell subset features an immunosuppressive property 18.

The number of mouse CD4^+^ NKG2D^+^ T cell population significantly increased in RAE‐1ε transgenic mice, whose RAE‐1ε expression was controlled by the CD86 promoter. CD4^+^ NKG2D^+^ T cells produced TGF‐β to down‐regulate NKG2D expression on NK cells, whereas Foxp3 was not expressed in the cytoplasm 19. Here, we investigated whether the regulatory CD4^+^ NKG2D^+^ T cells are associated with colitis induced by dextran sodium sulphate (DSS) in mice. Furthermore, whether the subsets of CD4^+^ NKG2D^+^ T cells with distinct function could be discriminated by additional cell markers remains unclear. Results show that the frequency of NK1.1^−^ CD4^+^ NKG2D^+^ T cells in colon is negatively correlated with colitis induced by DSS, and NK1.1^−^ CD4^+^ NKG2D^+^ T cell differs from NK1.1^+^ CD4^+^ NKG2D^+^ T cells in terms of cell membrane markers and transcriptional RNAs.

## Materials and methods

### Reagents and mice

The following antibodies were obtained from Biolegend (San Diego, CA) or eBioscience (San Diego, CA): CD3 (17A2), γδ (GL3), CD8 (53.67), CD4 (GK1.5), NK1.1 (PK136), NKG2D (CX5), CD107a (1D4B), IFN‐γ (XMG1.2), NKp46 (29A1.4), NKG2A (16A11), Ly49D (4E5), Ly49H (3D10), TGF‐β (TW7‐16B4), FasL (MFL3), IL‐10 (JES5‐16E3), IL‐17 (eBio17B7), CD62L (MEL‐14), CD44 (IM7), granzyme B (NG2B), perforin (eBioOMAK‐D), CD25 (PC61.5), Foxp3 (FJK‐16S), GITR (YGITR 765), CTLA‐4 (UC10‐4B9), CD39 (24DMS1), CD69 (LG.3A10), CCR9 (CW‐1.2), CD28 (E18), T‐bet (4B10), GATA‐3 (16E10A23) and ROR‐γt (AFKJS‐9), neutralized TGF‐β antibody (1D11) and RAE‐1ε mAb (205001). C57BL/6 and pCD86‐RAE‐1 transgenic mice 19 were generated and housed in accordance with the rules of Animal Committee of Yangzhou University.

### Induction and evaluation of acute colitis in mice

Colitis was induced by administration of DSS (2.5% w/v; m.w., 36–50 kD; MP Biomedicals, Santa Ana, CA, USA) to drinking water for 7 days (*n *= 5). All mice were weighed every day. To assess the extent of colitis, loss in bodyweight (0, none; 1, 1% to 5%; 2, 5% to 10%; 3, 10% to 20%; and 4, >20%), stool consistency (0, normal; 2, loose stools; and 4, watery diarrhoea) and blood in the stool (0, normal; 2, slight bleeding; and 4, gross bleeding) were monitored daily by trained individuals blinded to the treatment groups. Disease activity scores are calculated using the total score, which ranged from 0 to 12. The mice were killed on day 8, and the spleens and intestinal tissues were removed for *ex vivo* analysis. All experimental protocols were approved by the Institutional Animal Care and Use Committee of Yangzhou University.

### Isolation of colonic lymphocytes

Colon tissues of experimental mice were collected and washed completely with cold phosphate‐buffered saline (PBS). The tissues were dissected longitudinally, washed completely and cut into smaller pieces. The tissues were then predigested by Hanks’ balanced salt solution (HBSS) with 5 mM EDTA and 1 mM DTT at 37°C for 20 min. Mixed cell solution was passed through a nylon filter (100 μm) and then digested in PBS containing collagenase D (0.5 g/L), DNase I (0.5 g/L) and dispase II (3 g/L) for another 20 min. The cell suspension was centrifuged, suspended and washed with RPMI 1640 three times. The mixed cells were supplemented with 35% Percoll and then centrifuged to isolate mononuclear cells. Finally, the mononuclear cells were washed with PBS for further study.

### Flow cytometric intracellular staining

Cytokine production was determined using an intracellular staining kit (eBioscience). Lymphocytes were cultured with PMA (200 ng/ml)/ionomycin (2 μg/ml) in the presence of brefeldin A (10 μg/ml) for 4 hrs at 37°C. At the end of the incubation period, the cells were pre‐stained with antibody against surface markers. The cells were fixed, permeabilized, stained with cytokine or isotype antibody and analysed by flow cytometry. Lymphocytes were also permeabilized in referenced buffer and incubated with antibodies against Foxp3, T‐bet, GATA‐3 or ROR‐γt.

### Immunofluorescence

Mouse tissues were embedded in OCT and frozen instantly in liquid nitrogen for cryostat sections. After fixation, the sections were blocked with donkey serum and stained with goat antimouse RAE‐1ε antibody (AF1136; R&D systems, Minneapolis, MN, USA). The sections were washed and stained with Alexa‐546 labelled donkey anti‐goat as secondary antibody. The sections were covered with DAPI (Vector Labs, Burlingame, CA, USA). Fluorescence was detected using Eclipse E600 (Nikon, Japan) microscope and analysed using NIS‐Elements software (Nikon, Japan).

### Adoptive transfer of lymphocytes

Splenic NK1.1^−^ CD4^+^ cells from pCD86‐RAE‐1 transgenic mice were negatively isolated using a CD4^+^ T cell isolation kit. The cells were stained with PE‐antimouse NKG2D antibody and magnetic‐labelled anti‐PE antibody sequentially. The sorted CD4^+^ NKG2D^+^ T cells (5 × 10^5^) were injected into the tail vein of DSS‐ or PBS‐treated mice (*n *= 5) on days 1, 3 and 5. On day 7, the mice were killed to obtain mononuclear colon cells and spleens. Frequencies of CD4^+^ NKG2D^+^ T cells were analysed using flow cytometry gated on 7‐AAD^−^ CD45^+^ NK1.1^−^ cells.

### RNA profile analysis

Splenic NK1.1^−^ CD4^+^ NKG2D^+^ and NK1.1^+^ CD4^+^ NKG2D^+^ cells of normal C57BL/6 mice were sorted using flow cytometry. The sorted cells were lysed in TRIzol reagent (Invitrogen, Carlsbad, CA, USA) and reverse‐transcribed into complementary DNAs. The DNA profiles were detected by GeneChip^®^ Mouse Transcriptome Assay 1.0 (Affymetrix, Santa Clara, CA, USA) in Shanghai GIMIX Information Technology Company. Data were analysed by GCBI online software.

## Results

### Colonic NK1.1^−^ CD4^+^ NKG2D^+^ cells are negatively associated with DSS‐induced colitis

**Figure 1:**
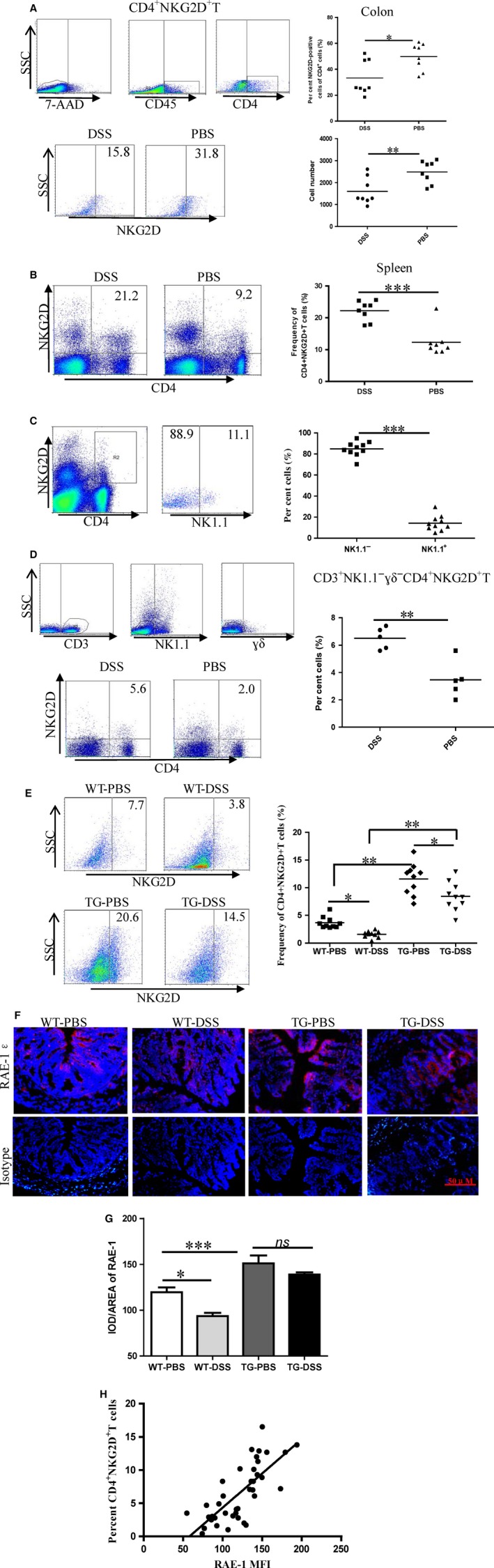
Decreased frequency of colonic NK1.1^−^ CD4^+^ NKG2D^+^ T cells in mice treated by DSS. (**A**) Mononuclear colon cells from DSS‐treated or control mice were separated and analysed on CD4^+^ NKG2D^+^ cells gated on 7‐AAD ^−^ CD45^+^ cells. Numbers of mononuclear cells were detected by cell counter. (**B**) Splenic frequency of CD4^+^ NKG2D^+^ cells was analysed by flow cytometry. (**C**) Splenic CD4^+^ NKG2D^+^ cells were stained by NK1.1 antibody. (**D**) Mononuclear cells from spleen were analysed on CD4^+^ NKG2D^+^ cell frequency gating on CD3^+^ γδ^−^ NK1.1^−^ cells. (**E**) Frequencies of colonic NK1.1‐CD4^+^ NKG2D^+^ T cells from pCD86‐RAE‐1 DSS‐treated transgenic or wild mice were detected. (**F**) Colon sections were stained by RAE‐1 antibody, and images were magnified 200‐fold. (**G**) The fluorescence was read by the NIS‐Elements software. (**H**) Linear regression analysis of RAE‐1 expression level of colon tissues with frequency of colonic NK1.1^−^ CD4^+^ NKG2D^+^ T cells (*r* ^2^ = 0.56, *P *< 0.001). All experiments were repeated at least three times.

Mononuclear colon cells from DSS‐ or PBS‐treated mice were isolated, and frequencies of 7‐AAD^−^ CD45^+^ CD4^+^ NKG2D^+^ cells were analysed. The frequency of CD4^+^ NKG2D^+^ cells was significantly down‐regulated in the local inflamed colons of DSS‐treated mice (Fig. 1A), whereas CD4^+^ NKG2D^+^ cells increased in the spleen of DSS‐treated mice (Fig. 1B). The absolute number of CD4^+^ NKG2D^+^ cells also decreased in the colons of DSS‐treated mice (Fig. 1A). More than 80% of CD4^+^ NKG2D^+^ T cells did not express NK1.1, which is an NK cell marker (Fig. 1C). However, the frequency of total CD4^+^ T cells was enhanced in the colons but decreased in the spleens of DSS‐treated mice compared with those in PBS‐treated mice (Supplementary Fig. 1A, 2A). As a result of inflammation, the frequencies of CD8^+^ T, CD8^+^ NKG2D^+^ T, NK1.1^+^, NK1.1^+^ NKG2D^+^ cells also increased in the colons of DSS‐treated mice (Supplementary Fig. 1B,C). No significant changes in CD8^+^ and NK1.1^+^ cell frequencies were observed in the spleen of DSS‐treated mice. However, CD8^+^ NKG2D^+^ and NK1.1^+^ NKG2D^+^ cell frequencies of spleens decreased in DSS‐treated mice (Supplementary Fig. 2B,C). Thus, the distribution of CD4^+^ NKG2D^+^ cells in the colons of DSS‐treated mice was distinct from that of conventional CD8^+^ NKG2D^+^ and NK1.1^+^ NKG2D^+^ cells with pro‐inflammatory activity.

Splenic cells were analysed on CD3^+^ NK1.1^−^ γδ^−^ CD4^+^ NKG2D^+^ cells to confirm variations in real CD4^+^ NKG2D^+^ T cells in the spleens of DSS‐treated mice. As predicted, splenic CD3^+^ NK1.1^−^ γδ^−^ CD4^+^ NKG2D^+^ cell frequency was enhanced in the DSS treatment group (Fig. 1D). Meanwhile, splenic CD3^+^ NK1.1^+^ γδ^−^ CD4^+^ NKG2D^+^ cell frequency decreased, suggesting that CD3^+^ NK1.1^−^ γδ^−^ CD4^+^ NKG2D^+^ cells play a different role from that of CD3^+^ NK1.1^+^ γδ^−^ CD4^+^ NKG2D^+^ cells (Supplementary Fig. 3).

Delayed onset of DSS‐induced colitis was observed in the pCD86‐RAE‐1 transgenic mice, in which numerous CD4^+^ NKG2D^+^ T cells were induced 19. After DSS treatment, we checked the frequencies of NK1.1^−^ CD4^+^ NKG2D^+^ cells in the colon of transgenic and wild mice. Compared with that in PBS treatment, the frequency of NK1.1^−^ CD4^+^ NKG2D^+^ cells in the colon decreased in transgenic DSS‐treated mice. However, the frequency of NK1.1^−^ CD4^+^ NKG2D^+^ cells in the colon of transgenic DSS‐treated mice remained higher than those in wild‐type DSS‐treated mice (Fig. 1E). The frequency of NK1.1^−^ CD4^+^ NKG2D^+^ cells in the spleen of transgenic DSS‐treated mice was also enhanced (Supplementary Fig. 4).

RAE‐1 expression in colon tissues was observed using an immunofluorescence technique to investigate whether the RAE‐1 expression level of intestinal epithelium is correlated with the infiltration of NK1.1^−^ CD4^+^ NKG2D^+^ cells into the colon. RAE‐1 expression level decreased in the colon epithelium of wild‐type DSS‐treated mice but was maintained in the colons of transgenic DSS‐treated mice (Fig. 1F,G). A positive correlation was found between RAE‐1 expression level and frequency of infiltrated NK1.1^−^ CD4^+^ NKG2D^+^ cells in the colon (*r* ^2^ = 0.56, *P *< 0.001) (Fig. 1H). RAE‐1 expression level in the colon epithelium affected the recruitment of NK1.1^−^ CD4^+^ NKG2D^+^ cells.

### NK1.1^−^CD4^+^ NKG2D^+^ T cells produce TGF‐β without cytotoxicity

**Figure 2:**
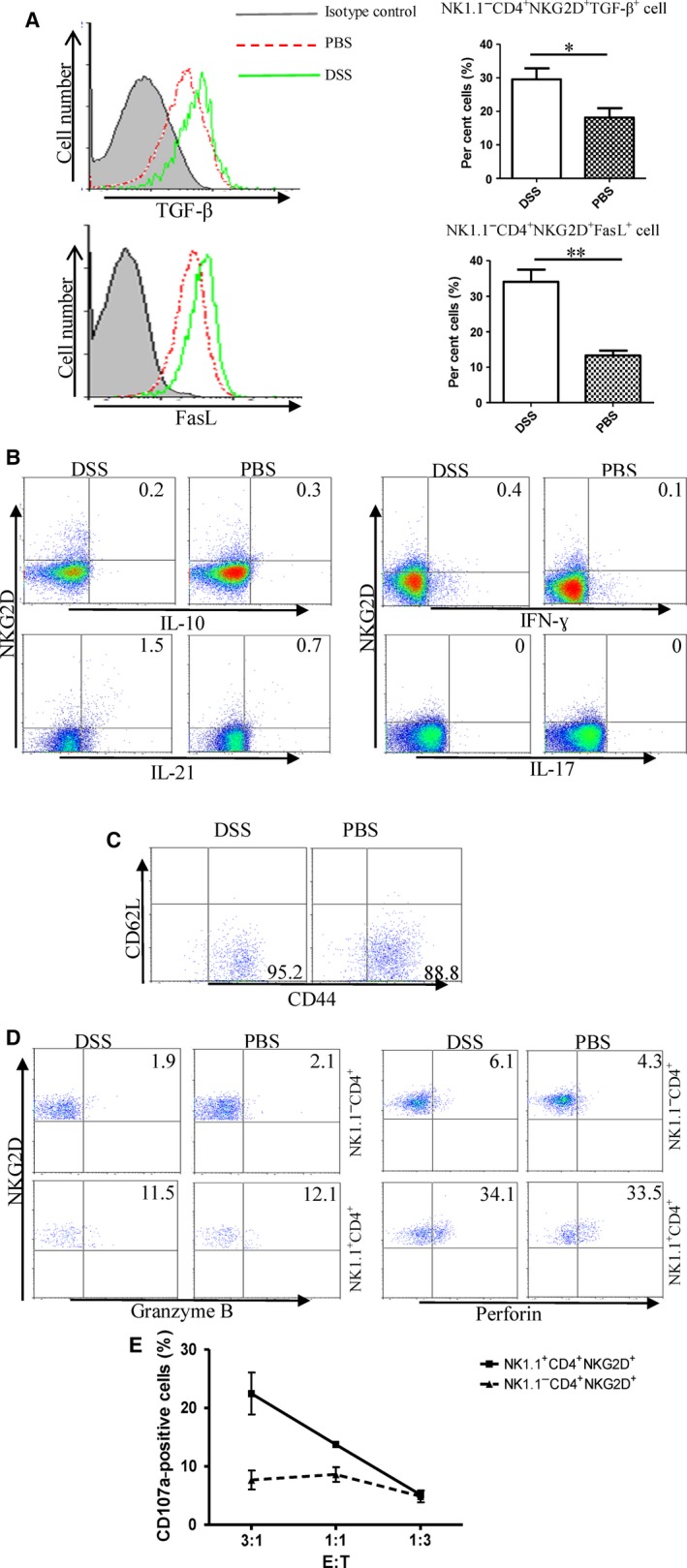
Capacity of cytokine production and cytotoxicity of NK1.1^−^ CD4^+^ NKG2D^+^ cells. (**A**) NK1.1^−^ CD4^+^ NKG2D^+^ cells were stained by TGF‐β and FasL antibody. (**B**) NK1.1^−^ CD4^+^ NKG2D^+^ cells were intracellularly stained by IL‐10, IFN‐γ, IL‐21 and IL‐17 antibodies after stimulation by PMA and ionomycin. (**C**) Costaining of CD62L and CD44 on NK1.1^−^ CD4^+^ NKG2D^+^ cells. (**D**) Intracellular staining of granzyme B and perforin. (**E**) Degranulation of NK1.1^−^ CD4^+^ NKG2D^+^ and NK1.1^+^ CD4^+^ NKG2D^+^ cell after incubating with B16‐MICA target cells is measured by CD107a expression on cell membrane. All experiments were performed three times.

We analysed the capability of cells to produce cytokines and mediate cytotoxicity. The subpopulation of NK1.1^−^ CD4^+^ NKG2D^+^ T cells from both DSS‐treated and control mice highly expressed TGF‐β and FasL. Simultaneously, DSS‐treated mice exhibited increased frequencies of NK1.1^−^ CD4^+^ NKG2D^+^ TGF‐β^+^ and NK1.1^−^ CD4^+^ NKG2D^+^ FasL^+^ cells in the spleen compared with those in PBS‐treated mice (Fig. 2A). The subset of NK1.1^−^ CD4^+^ NKG2D^+^ cells did not secrete IL‐10, in contrast to conventional regulatory T cells. In addition, NK1.1^−^ CD4^+^ NKG2D^+^ cells could almost not produce IFN‐γ, IL‐21 and IL‐17 (Fig. 2B), whereas NK1.1^+^ CD4^+^ NKG2D^+^ cells produced IFN‐γ, IL‐21 and IL‐17 (Supplementary Fig. 5). Phenotypic analysis showed that most NK1.1^−^ CD4^+^ NKG2D^+^ cells were CD62L^−^ CD44^+^, suggesting that the subpopulation was in activated state (Fig. 2C).

Although NK1.1^+^ CD4^+^ NKG2D^+^ cells expressed granzyme B and perforin in the cytoplasm, NK1.1^−^ CD4^+^ NKG2D^+^ cells almost negatively expressed cytolytic granules (Fig. 2D). NK1.1^−^ CD4^+^ NKG2D^+^ or NK1.1^+^ CD4^+^ NKG2D^+^ cells were incubated with melanoma target cells, B16‐MICA, which ectopically expressed MICA of B16 cells. NK1.1^+^ CD4^+^ NKG2D^+^ cells displayed significant degranulation, in contrast to NK1.1^−^ CD4^+^ NKG2D^+^ cells (Fig. 2E). Therefore, CD4^+^ NKG2D^+^ cells could be further divided into two subsets based on NK1.1 expression. NK1.1^+^ CD4^+^ NKG2D^+^ cells may function as NK cell‐like CD4^+^ T cells, and NK1.1^−^ CD4^+^ NKG2D^+^ cells mediate regulatory functions by producing TGF‐β and FasL.

### Adoptive transfer of CD4^+^ NKG2D^+^ NK1.1^−^ T cells suppresses the onset of colitis induced by DSS

**Figure 3:**
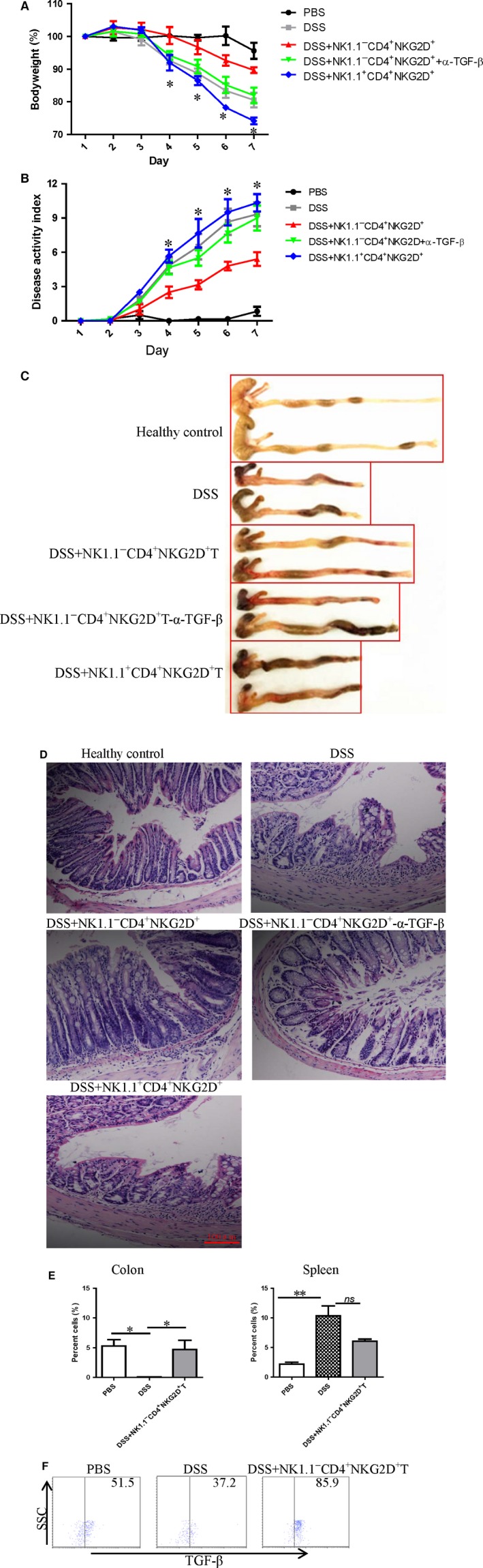
Adoptive transfer of NK1.1^−^ CD4^+^ NKG2D^+^ cells suppressed DSS‐induced colitis. (**A**) Weight curve of DSS‐mice treated with NK1.1^−^ CD4^+^ NKG2D^+^ cells, NK1.1^+^ CD4^+^ NKG2D^+^ cells or NK1.1^−^ CD4^+^ NKG2D^+^ cells pre‐incubated with TGF‐β antibody. (**B**) Disease activity index of all groups of mice with colitis. (**C**) Morphological changes of colons with a variety of stimulations. * represents comparison between the DSS‐treated group and the NK1.1^−^ CD4^+^ NKG2D^+^ cell transferred DSS‐treated group. (**D**) Histology of colon sections in various treatments (×200). (**E**) CD4^+^ NKG2D^+^ T cells sorted by magnetic‐labelled antibody were injected into the tail veins of DSS‐treated mice on days 1, 3 and 5. On day 7, the mice were killed to obtain mononuclear cells. The frequency of 7‐AAD ^−^ CD45^+^ NK1.1^−^ CD4^+^ NKG2D^+^ cells was analysed. (**F**) TGF‐β staining of colonic NK1.1^−^ CD4^+^ NKG2D^+^ cells. The experiment was repeated thrice.

Splenic NK1.1^−^ CD4^+^ NKG2D^+^ cells from healthy mice were isolated and adoptively transfused to the tail veins of mice pre‐treated with DSS to verify whether NK1.1^−^ CD4^+^ NKG2D^+^ cells can mediate immune regulation. As predicted, DSS‐treated mice transfused with NK1.1^−^ CD4^+^ NKG2D^+^ cells delayed the onset of colitis and decreased disease severity (Fig. 3A,B). Some regulatory T cells also express membrane‐bound TGF‐β 20. Membrane TGF‐β expression on fresh NK1.1^−^ CD4^+^ NKG2D^+^ cells of spleens from DSS‐ or PBS‐treated mice was confirmed by flow cytometric analysis without cell permeabilization (Supplementary Fig. 6). When NK1.1^−^ CD4^+^ NKG2D^+^ cells were pre‐incubated with TGF‐β antibody *in vitro*, the protective effect against colitis by adoptive transfusion almost disappeared. Thus, TGF‐β produced by NK1.1^−^ CD4^+^ NKG2D^+^ cells was considered a key effector molecule in the local environment.

Severity of colitis in mice was confirmed by visible inflamed colons and histological analysis on the colon sections. The shortened length of colons in mice induced by DSS was elongated by transfusing with NK1.1^−^ CD4^+^ NKG2D^+^ cells. However, transfer of NK1.1^+^ CD4^+^ NKG2D^+^ cells exacerbated colitis in mice, because of more shortened length and severe haemorrhage of colons (Fig. 3C). TGF‐β blocking almost diminished the protective effect (Fig. 3C). In addition, the colon sections of mice transfused with NK1.1^−^ CD4^+^ NKG2D^+^ cells showed less infiltration of mononuclear cells and had more regular villa. The colon sections from TGF‐β blockage and NK1.1^+^ CD4^+^ NKG2D^+^ cell transfer group showed irregular villa and signs of mononuclear cell infiltration (Fig. 3D). The frequency of NK1.1^−^ CD4^+^ NKG2D^+^ cells of colons significantly increased in DSS‐treated mice with adoptive transfer of NK1.1^−^ CD4^+^ NKG2D^+^ cells. However, no significant variations in NK1.1^−^ CD4^+^ NKG2D^+^ cell frequency were detected in the spleen of DSS‐treated mice with or without the transfer of NK1.1^−^ CD4^+^ NKG2D^+^ cells (Fig. 3E). In addition, colonic NK1.1^−^ CD4^+^ NKG2D^+^ cells also highly expressed TGF‐β, as confirmed in Fig. 3F.

### NK1.1^−^ CD4^+^ NKG2D^+^ T cells are phenotypically distinct from CD4^+^ CD25^+^ Foxp3^+^ regulatory cells

**Figure 4:**
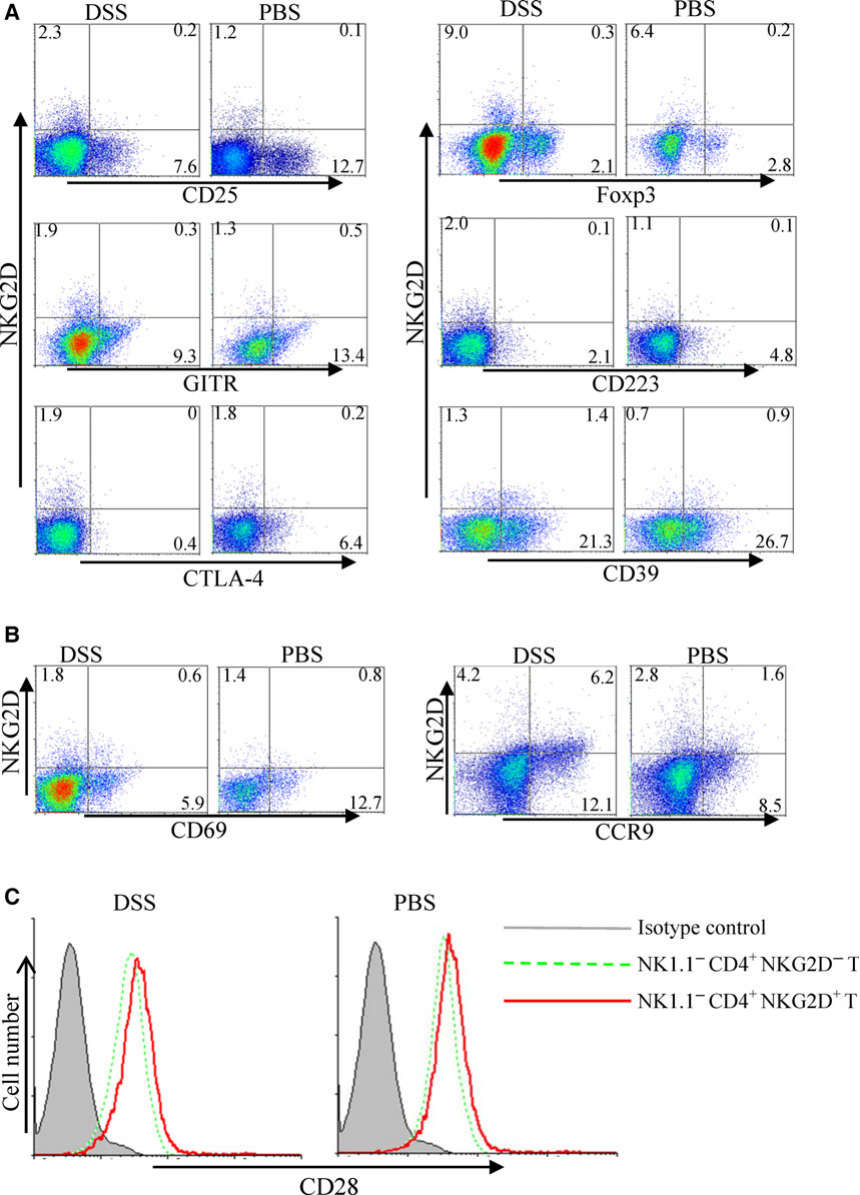
Comparison of phenotypic markers between splenic NK1.1^−^ CD4^+^ NKG2D^+^ and CD4^+^ CD25^+^ Foxp3^+^ cells. CD25, Foxp3, GITR, CD223, CTLA‐4, CD39 (**A**), CD69 and CCR9 (**B**) expression of NK1.1^−^ CD4^+^ NKG2D^+^ cells from DSS‐ or PBS‐treated mice. (**C**) CD28 expression on NK1.1^−^ CD4^+^ NKG2D^+^ and NK1.1^−^ CD4^+^ NKG2D^−^ cells. The experiment was repeated at least six times.

We discriminated NK1.1^−^ CD4^+^ NKG2D^+^ T cells with regulatory function from conventional CD4^+^ CD25^+^ Foxp3^+^ regulatory T cells. NK1.1^−^ CD4^+^ NKG2D^+^ T subpopulation of spleens from both DSS‐treated and control mice did not express CD25, Foxp3, GITR, CD223 (LAG‐3) and CTLA‐4, but expressed CD39 at low levels (Fig. 4A). These cell markers are regarded as Treg‐associated molecules (Supplementary Fig. 7) 21, 22. NK1.1^−^ CD4^+^ NKG2D^+^ T cells moderately expressed CD69 and chemotactic receptor CCR9 (Fig. 4B), indicating that these cells exhibit potential to migrate into the intestine. Several studies show that CD4^+^ NKG2D^+^ T cells with CD28 negative expression have autoreactivity and promote local inflammation 23, 24. In the present study, NK1.1^−^ CD4^+^ NKG2D^+^ T cells increased CD28 expression level compared with NK1.1^−^ CD4^+^ NKG2D^−^ T cells in both DSS‐treated and control mice. NK1.1^−^ CD4^+^ NKG2D^+^ T cells differed from inflammatory CD4^+^ NKG2D^+^ T cell subset and conventional CD4^+^ CD25^+^ Foxp3^+^ regulatory T cell (Fig. 4C).

### NK1.1^−^ CD4^+^ NKG2D^+^ T cells are phenotypically different from NK1.1^+^ CD4^+^ NKG2D^+^ cells

**Figure 5:**
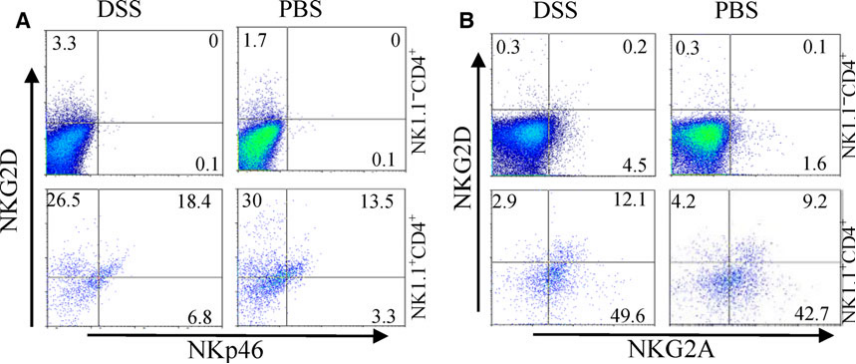
Expressions of NK cell receptors of NK1.1^−^ CD4^+^ NKG2D^+^ and NK1.1^+^ CD4^+^ NKG2D^+^ cells. NKp46 (**A**) and NKG2A (**B**) expression was detected using flow cytometry gated on NK1.1^−^ CD4^+^ and NK1.1^+^ CD4^+^ cells. The experiment was repeated at least six times.

Under some conditions, T cells can be reprogrammed to express NK cell receptor particularly after stimulation of IL‐15 25, 26. We investigated the expression of NK cell receptors in NK1.1^−^CD4^+^ NKG2D^+^ and NK1.1^+^ CD4^+^ NKG2D^+^ cells. Very low or no expression of NKp46 (Fig. 5A) and NKG2A (Fig. 5B) was observed in NK1.1^−^ CD4^+^ NKG2D^+^ T cells, whereas both receptors were expressed in NK1.1^+^ CD4^+^ NKG2D^+^ cells. Thus, NK1.1^−^ CD4^+^ NKG2D^+^ T cells did not express other NK cell receptors and were phenotypically different from NK‐like CD4^+^T cells.

### Comparison of transcriptional RNAs of NK1.1^−^CD4^+^NKG2D^+^T and NK1.1^+^CD4^+^NKG2D^+^T cells

**Figure 6:**
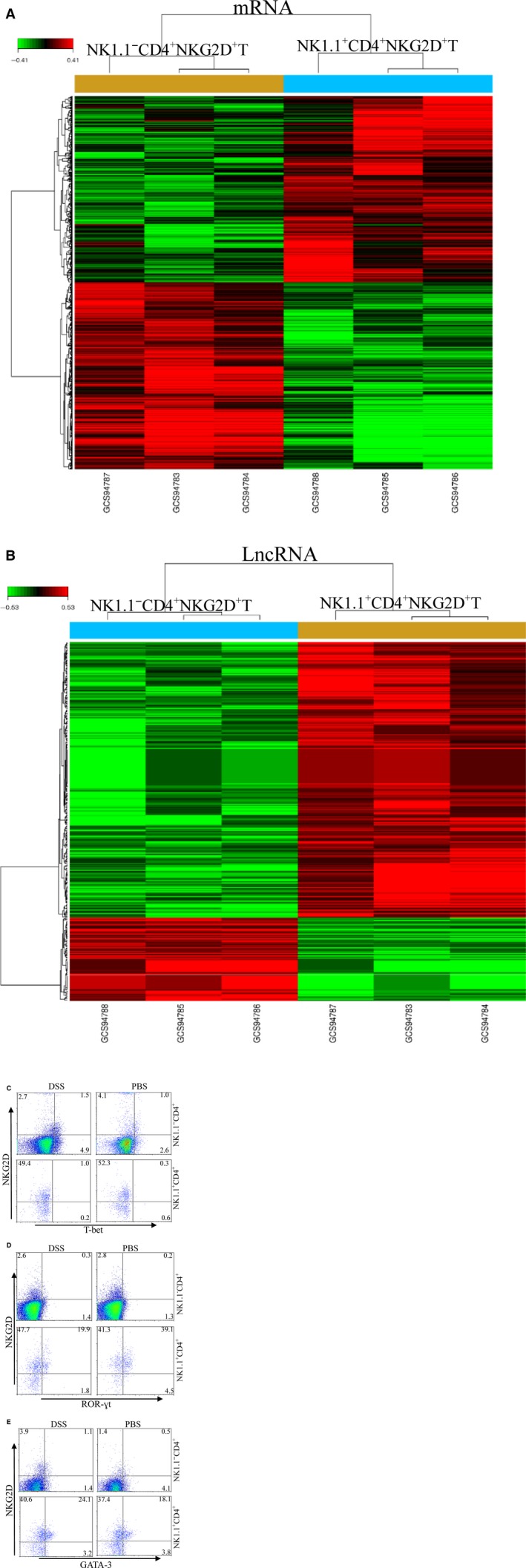
Comparison of RNA transcription profiles between NK1.1^−^ CD4^+^ NKG2D^+^ and NK1.1^+^ CD4^+^ NKG2D^+^ cells. Gene array analysis of mRNA (**A**) and lncRNA (**B**). Two subpopulations were isolated from spleens of normal mice. Intracellular T‐bet (**C**), ROR‐γt (**D**) and GATA‐3 (**E**) were detected using flow cytometry gated on NK1.1^−^ CD4^+^ and NK1.1^+^ CD4^+^ cells. The experiment was repeated three times.

We further compared the transcriptional RNAs of NK1.1^+^ CD4^+^ NKG2D^+^ T and NK1.1^−^CD4^+^ NKG2D^+^ T cells. NK1.1^+^ CD4^+^ NKG2D^+^ T and NK1.1^−^ CD4^+^ NKG2D^+^ T cells were purified using flow cytometry, and total RNA was isolated. The mRNA and long non‐coding RNA (lncRNA) profiles were analysed by gene chips. A total of 161 mRNA genes (Fig. 6A) and 741 lncRNA (Fig. 6B) genes (threshold = 2) were altered between the two subsets. These results suggested that NK1.1^+^ CD4^+^ NKG2D^+^ T and NK1.1^−^ CD4^+^ NKG2D^+^ T cells were absolutely distinct.

Cytosolic T‐bet, GATA‐3 and ROR‐γt were detected in NK1.1^+^ CD4^+^ NKG2D^+^ T and NK1.1^−^ CD4^+^ NKG2D^+^ T cells to investigate nuclear factors associated with differentiation of NK1.1^−^ CD4^+^ NKG2D^+^ T cells. NK1.1^−^ CD4^+^ NKG2D^+^ T cells moderately expressed T‐bet, but NK1.1^+^ CD4^+^ NKG2D^+^ T cells very lowly expressed T‐bet (Fig. 6C). Although no ROR‐γt expression was found in NK1.1^−^ CD4^+^ NKG2D^+^ T cells, ROR‐γt was highly expressed in NK1.1^+^ CD4^+^ NKG2D^+^ T cells (Fig. 6D). In addition, NK1.1^−^ CD4^+^ NKG2D^+^ T cells expressed low levels of GATA‐3 in the cytoplasm, and NK1.1^+^ CD4^+^ NKG2D^+^ T cells showed enhanced expression of GATA‐3 (Fig. 6E). Thus, NK1.1^−^ CD4^+^ NKG2D^+^ T cells could not differentiate into Th2 and Th17 cells. The subset performed regulatory functions with expression of T‐bet.

## Discussion

In this work, we divided CD4^+^ NKG2D^+^ T cells into two subsets based on NK1.1 expression. Colonic NK1.1^−^ CD4^+^ NKG2D^+^ T cells were inversely correlated with DSS‐induced colitis in mice. NK1.1^−^ CD4^+^ NKG2D^+^ T cells highly produced TGF‐β and FasL, expressed IL‐10, IFN‐γ, IL‐21 and IL‐17 at low levels and had no cytotoxicity against target cells. Adoptive transfer of NK1.1^−^ CD4^+^ NKG2D^+^ T cells into DSS‐treated mice suppressed the onset of colitis dependent on TGF‐β. NK1.1^−^ CD4^+^ NKG2D^+^ T cells did not express Foxp3 and receptors, which were conventionally expressed in NK cells. The pattern of mRNA and lncRNA transcription was distinct between NK1.1^−^ CD4^+^ NKG2D^+^ T and NK1.1^+^ CD4^+^ NKG2D^+^ T cells. We confirmed that NK1.1^−^ CD4^+^ NKG2D^+^ T cells are a special subset that mediated immune regulatory functions and are completely different from CD4^+^ CD25^+^ Foxp3^+^ regulatory T cells or NK1.1^+^ CD4^+^ NKG2D^+^ T cells, which could be involved in inflammation.

CD4^+^ T cells with NKG2D positive expression were reported in patients bearing tumour or inflammation‐associated diseases. CD4^+^ NKG2D^+^ T cells with regulatory function were found in both MICA^+^ cancer patients 18 and juvenile‐onset systemic lupus patients 17. Expression of NK cell markers by those CD4^+^ NKG2D^+^ T cells remains unclear. NK1.1^−^ CD4^+^ NKG2D^+^ mouse cells expressed CD28 at higher levels than that of conventional CD4^+^ NKG2D^−^ effector T cells. NK1.1^−^ CD4^+^ NKG2D^+^ CD28^high^ cells were different from CD4^+^ NKG2D^+^ CD28^−^ cells involved with inflammation. Compared with conventional CD4^+^ CD25^+^ Foxp3^+^ regulatory T cells, NK1.1^−^ CD4^+^ NKG2D^+^ T cells did not express CD25, Foxp3, GITR, CD223 (LAG‐3) 27 and CTLA‐4. The induction of regulatory CD4^+^ NKG2D^+^ cells was determined by costimulation of the MHC II molecule and NKG2D ligand 17, 18. Molecular mechanisms of TGF‐β production by NK1.1^−^ CD4^+^ NKG2D^+^ cell after coligation of TCR and NKG2D are still being studied.

The pro‐inflammatory role of CD4^+^ NKG2D^+^ T cells was observed in patients with rheumatoid arthritis 5, 6, multiple sclerosis 11, Crohn's disease 8, 9, 10, granulomatosis with polyangiitis 7 and CMV infection 12. In addition, CD4^+^ NKG2D^+^ T cells were negative in grade 1 cervical intraepithelial neoplasia and melanoma patients treated with sorafenib 16. CD161, a homolog of mouse NK1.1, is expressed on CD4^+^ NKG2D^+^ T cells of patients with Crohn's disease. Th17 and Th1 represent 30% ± 17% and 12% ± 5%, respectively, of the lamina propria CD4^+^ NKG2D^+^ population 9. In the present study, NK1.1^+^ CD4^+^ NKG2D^+^ cells produced IFN‐γ, IL‐17 and IL‐21, indicating that the NK1.1^+^ CD4^+^ NKG2D^+^ subset may resemble human CD161^+^ CD4^+^ NKG2D^+^ cells involved with inflammation 28, 29.

NK1.1^−^ CD4^+^ NKG2D^+^ cells showed distinctive markers from NK1.1^+^ CD4^+^ NKG2D^+^ cells. NKp46 30 and NKG2A 31 were not expressed in NK1.1^−^ CD4^+^ NKG2D^+^ cells. Given that IL‐15 stimulations can program T cells to express NK cell markers, such as NKp46 and NKG2A 5, 7, 15, the induction and proliferation of NK1.1^−^ CD4^+^ NKG2D^+^ cells may not depend on IL‐15 alone. Induction of the subset *in vitro* requires coligations of TCR and NKG2D. Naturally occurring regulatory CD4^+^ NKG2D^+^ cells were also found in humans 16. Further studies must determine whether mouse NK1.1^−^ CD4^+^ NKG2D^+^ cells could be produced in thymus innately or induced outside.

The infiltration of NK1.1^−^ CD4^+^ NKG2D^+^ cells into the colon with colitis decreased, whereas the frequency of NK1.1^−^ CD4^+^ NKG2D^+^ cells in the spleen of mice was enhanced. Under inflammatory conditions, NK1.1^−^ CD4^+^ NKG2D^+^ cells would be redistributed because of the local environment. NK1.1^−^ CD4^+^ NKG2D^+^ T cells expressed low levels of CD39 32 and moderate levels of CD69 33 and CCR9 34, suggesting that NK1.1^−^ CD4^+^ NKG2D^+^ T cells exhibit potential to migrate into colon tissues. The retention of NK1.1^−^ CD4^+^ NKG2D^+^ T cells in spleens partly resulted from the enhanced expression level of NKG2D ligands of splenic macrophages or DCs under the DSS‐induced inflammatory situations. Macrophages or DCs would up‐regulate NKG2D ligand expression when they are stimulated by Toll‐like receptor ligand 35 or IFN‐α 36.The expression of the NKG2D ligand in islets is sufficient to recruit CTLs, regardless of their antigen specificity 37. RAE‐1 in colon epithelium also affected the recruitment of NK1.1^−^ CD4^+^ NKG2D^+^ T cells, because pCD86‐RAE‐1 transgenic mice showed higher expression levels of RAE‐1 in the colons after DSS treatment than those of the wild mice.

In conclusion, CD4^+^ NKG2D^+^ T cells were further categorized into NK1.1^−^ CD4^+^ NKG2D^+^ T and NK1.1^+^ CD4^+^ NKG2D^+^ T subsets. NK1.1^−^ CD4^+^ NKG2D^+^ T cells suppressed colitis induced by DSS in mice *via* production of TGF‐β. Regulatory NK1.1^−^ CD4^+^ NKG2D^+^ T cells differ from conventional CD4^+^ CD25^+^ Foxp3^+^ cells and do not have features of NK1.1^+^ CD4^+^ NKG2D^+^ T cells in both phenotype and RNA transcription. This article provides biological features of regulatory NK1.1^−^ CD4^+^ NKG2D^+^ T cells of mice and indicated that NK1.1^−^ CD4^+^ NKG2D^+^ T cells may be used for treatment of inflammation‐associated diseases.

## Disclosures

All authors have declared there are no financial conflicts of interest with regard to this work.

## Supporting information


**Figure S1** Detection of colonic T and NK cells of mice treated by DSS or PBS.
**Figure S2** Detection of splenic T and NK cells of mice treated by DSS or PBS.
**Figure S3** Detection of splenic CD3^+^ ɣδ^−^ NK1.1^+^ CD4^+^ NKG2D^+^ T of mice treated by DSS or PBS.
**Figure S4** Expression of Foxp3 in splenic NK1.1^−^CD4^+^NKG2D^+^ Foxp3^+^ cells of CD86‐transgenic or wild type mice treated by DSS.
**Figure S5** Production of IFN‐ɣ, IL‐17, and IL‐21 by NK1.1^+^ CD4^+^NKG2D^+^ cells of mice treated by DSS or PBS.
**Figure S6** Membrane TGF‐β on NK1.1^−^CD4^+^NKG2D^+^ cells of spleens from mice treated by DSS or PBS.
