# Supplementary material for: NK1.1− CD4+ NKG2D+ T cells suppress DSS‐induced colitis in mice through production of TGF‐β
Source: J Cell Mol Med. 2017 Feb 22;21(7):1431–44. doi: 10.1111/jcmm.13072 (PMC5487917; doi:10.1111/jcmm.13072)
Supplement: Supplementary file 1 — Figure S1 Detection of colonic T and NK cells of mice treated by DSS or PBS. Figure S2 Detection of splenic T and NK cells of mice treated by DSS or PBS. Figure S3 Detection of splenic CD3+ ɣδ− NK1.1+ CD4+ NKG2D+ T of mice treated by DSS or PBS. Figure S4 Expression of Foxp3 in splenic NK1.1−CD4+NKG2D+ Foxp3+ cells of CD86‐transgenic or wild type mice treated by DSS. Figure S5 Production of IFN‐ɣ, IL‐17, and IL‐21 by NK1.1+ CD4+NKG2D+ cells of mice treated by DSS or PBS. Figure S6 Membrane TGF‐β on NK1.1−CD4+NKG2D+ cells of spleens from mice treated by DSS or PBS. [file JCMM-21-1431-s001.pdf]

Supplementary Fig. 1

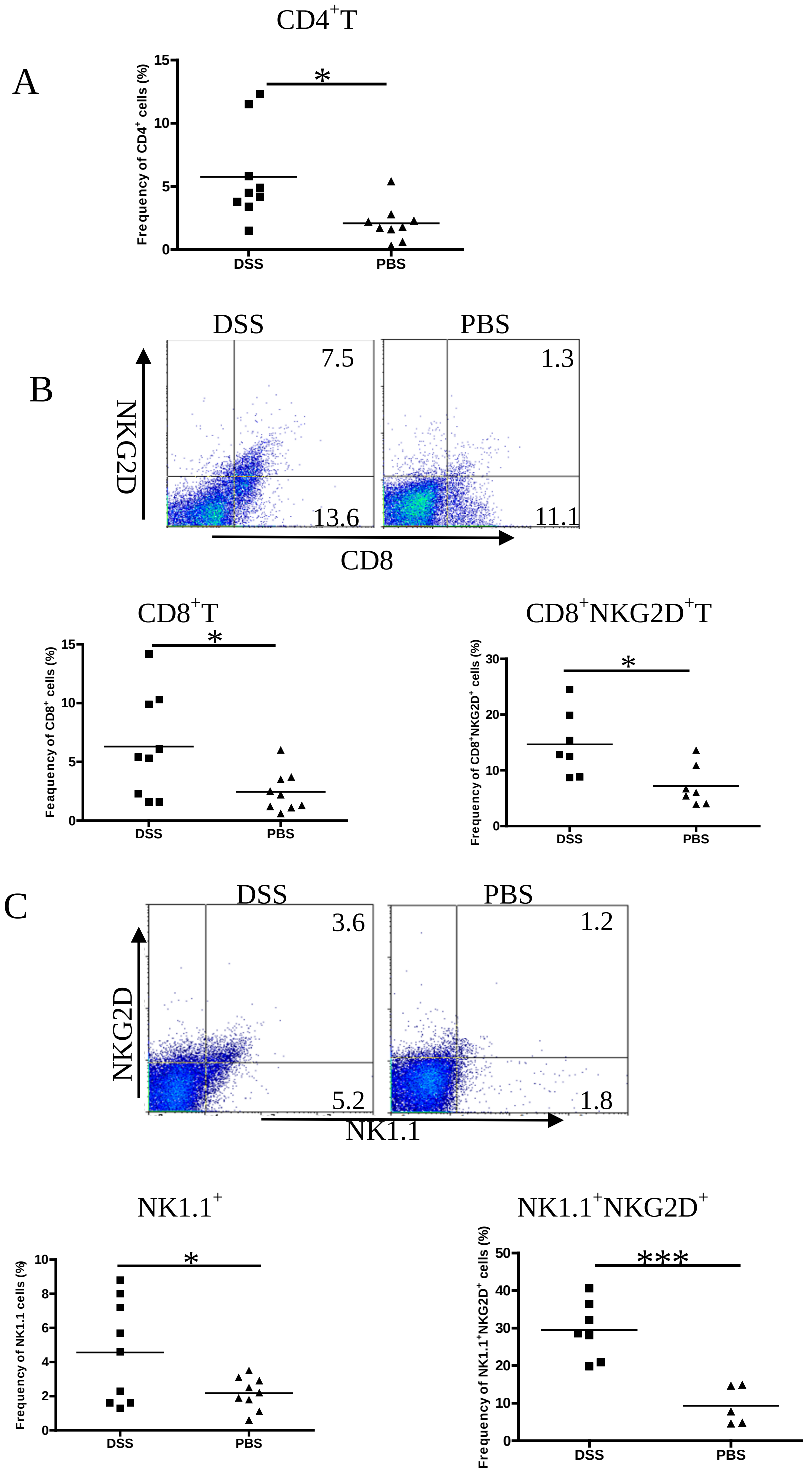

Supplementary figure 1. Detection of colonic T and NK cells of mice treated by DSS or PBS. (a) $CD4^+$ cell frequencies in colons of mice. (b) Frequencies of $CD8^+$ and $CD8^+$ $NKG2D^+$ cells in colons of mice. The upper panel is a representative result of $CD8^+$ $NKG2D^+$ cells of colons detected by flow cytometry. (c) Frequencies of $NK1.1^+$ and $NK1.1^+$ $NKG2D^+$ cells in colons of mice. The upper panel is a representative result of $NK1.1^+$ $NKG2D^+$ cells of colons detected by flow cytometry.

Supplementary Fig. 2

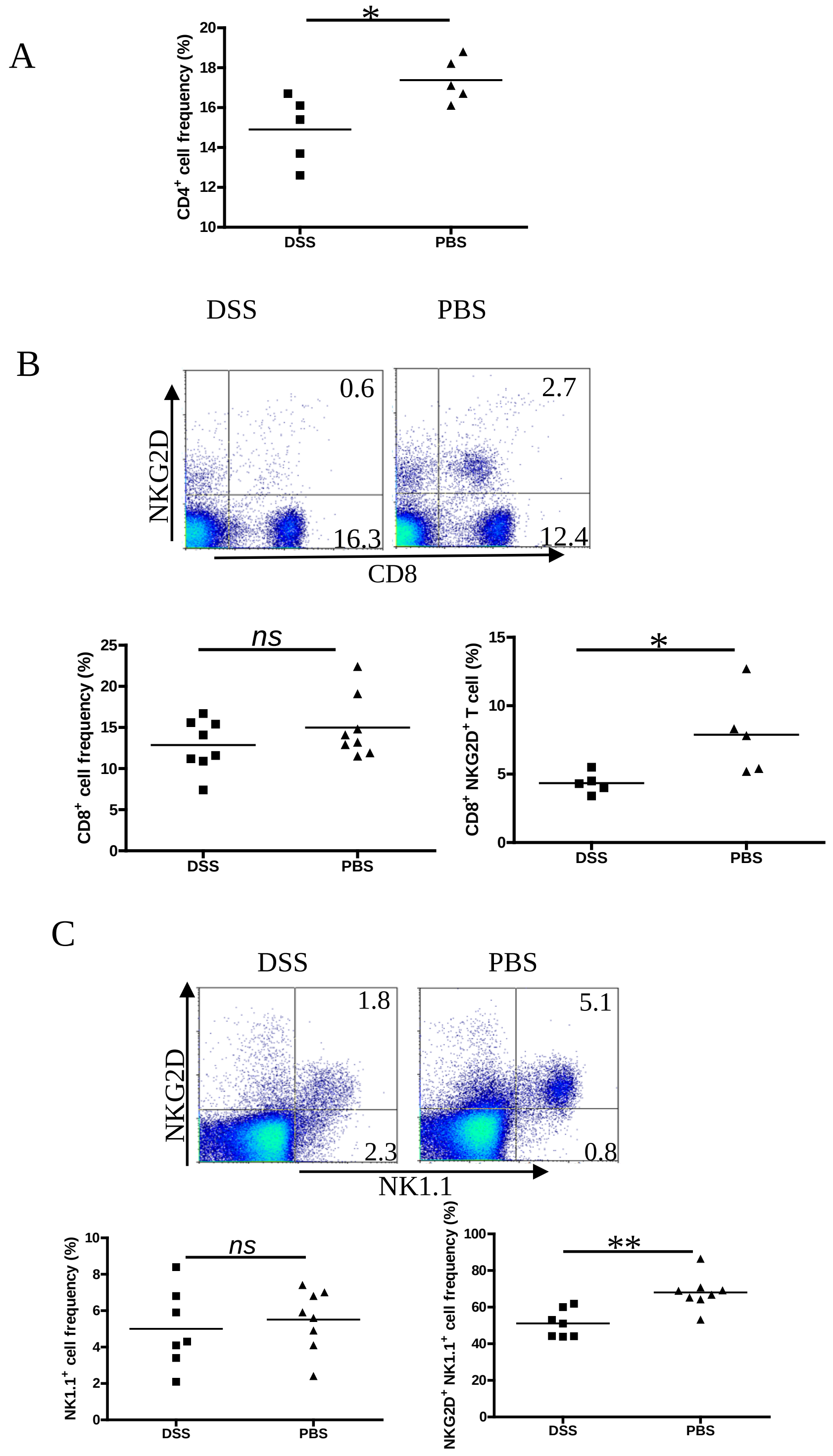

Supplementary figure 2. Detection of splenic T and NK cells of mice treated by DSS or PBS. (a) $CD4^+$ cell frequencies in spleens of mice. (b) Frequencies of $CD8^+$ and $CD8^+$ $NKG2D^+$ cells in spleens of mice. The upper panel is a representative result of $CD8^+$ $NKG2D^+$ cells of colons detected by flow cytometry. (c) Frequencies of $NK1.1^+$ and $NK1.1^+$ $NKG2D^+$ cells in spleens of mice. The upper panel is a representative result of $NK1.1^+$ $NKG2D^+$ cells of spleens detected by

flow cytometry.

Supplementary Fig.3

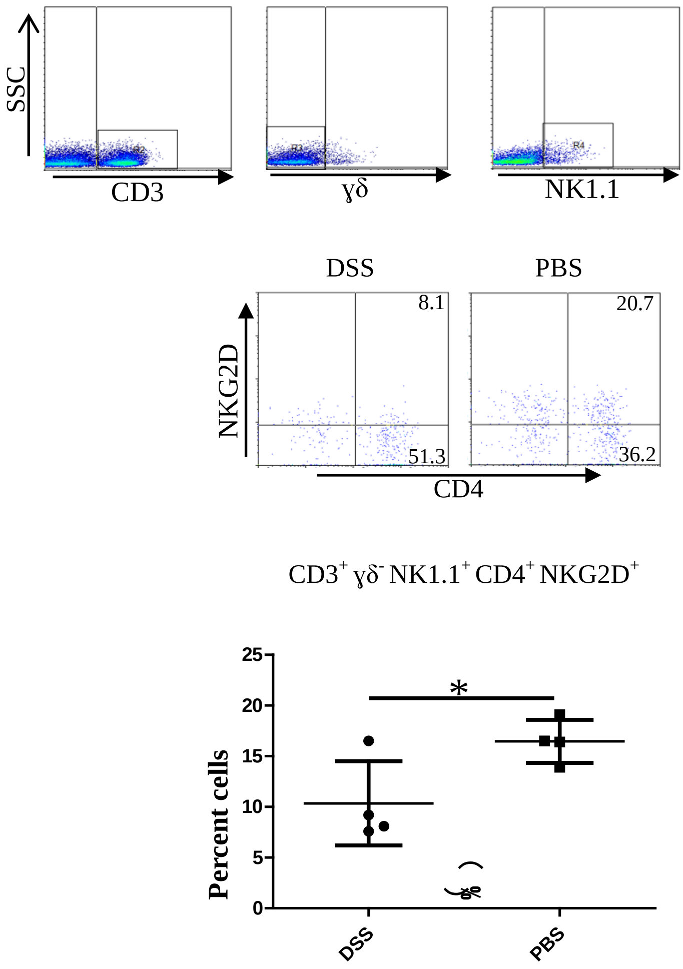

Supplementary figure 3. Detection of splenic $CD3^{+}$ $\gamma\delta^{-}$ $NK1.1^{+}$ $CD4^{+}$ $NKG2D^{+}$ T of mice treated by DSS or PBS. The upper panel is a representative result of lymphocytes detected by flow cytometry.

Supplementary Fig.4

Gated on NK1.1$^-$ CD4$^+$ cells

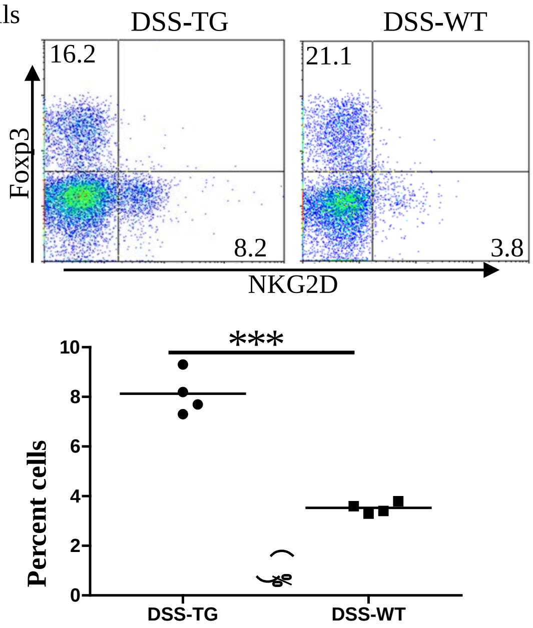

Supplementary figure 4. Expression of Foxp3 in splenic NK1.1$^-$ CD4$^+$ NKG2D$^+$ Foxp3$^+$ cells of CD86-transgenic or wild-type mice treated by DSS.

Supplementary Fig.5

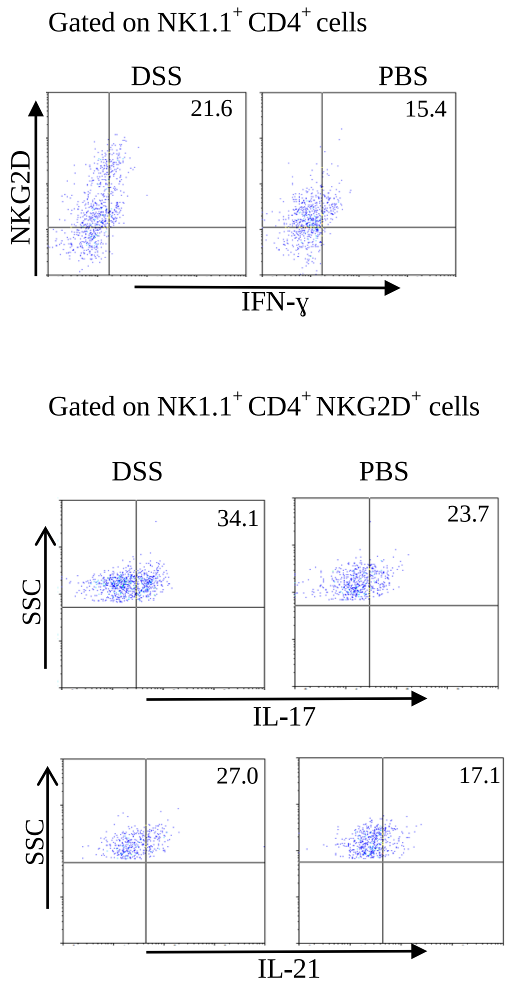

Supplementary figure 5. Production of IFN-γ, IL-17, and IL-21 by NK1.1^{+} CD4^{+} NKG2D^{+} cells of mice treated by DSS or PBS.

Supplementary Fig. 6

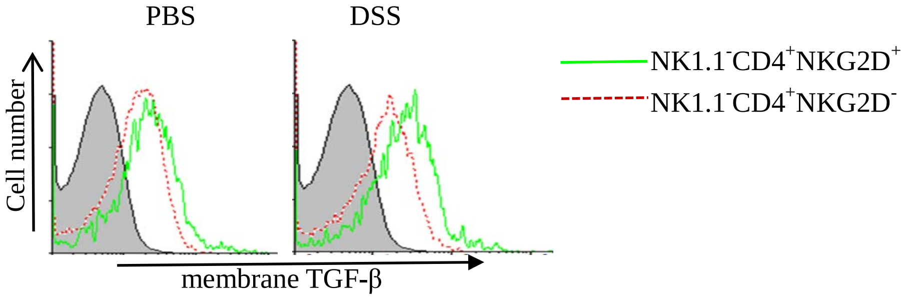

Supplementary figure 6. Membrane TGF-β on NK1.1$^-$CD4$^+$NKG2D$^+$ cells of spleens from mice treated by DSS or PBS.

Supplementary Fig.7

Gated on CD4$^+$ CD25$^+$ cells

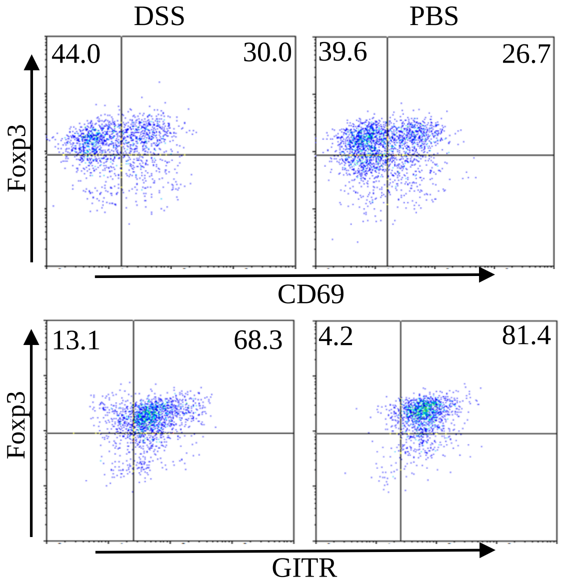

Supplementary figure 7. Expression of CD69 and GITR by CD4$^+$ CD25$^+$ Foxp3$^+$ cells of mice treated by DSS or PBS.
